# Pyrethroid resistance in the New World malaria vector *Anopheles albimanus* is mediated by cytochrome P450 *CYP6P5*

**DOI:** 10.1016/j.pestbp.2022.105061

**Published:** 2022-05

**Authors:** Michael O. Kusimo, Lucy Mackenzie-Impoinvil, Sulaiman S. Ibrahim, Abdullahi Muhammad, Helen Irving, Jack Hearn, Audrey E. Lenhart, Charles S. Wondji

**Affiliations:** a LSTM Research Unit, Centre for Research in Infectious Diseases (CRID), P.O. Box 13591, Yaoundé, Cameroon; b Entomology Branch, Division of Parasitic Diseases and Malaria, Centre for Global Health, Centres for Disease Control and Prevention, 1600 Clifton Rd, Atlanta, GA 30329, USA; c Vector Biology Department, Liverpool School of Tropical Medicine (LSTM), Liverpool L3 5QA, UK; d Department of Biochemistry, Bayero University, PMB 3011 Kano, Nigeria; e Centre for Biotechnology Research, Bayero University, PMB 3011 Kano, Nigeria

**Keywords:** *Anopheles albimanus*, *CYP6P5*, Metabolic, Resistance, α-Cypermethrin

## Abstract

Pyrethroid resistance in the malaria vector *Anopheles albimanus* presents an obstacle to malaria elimination in the Americas. Here, *An. albimanus CYP6P5* (the most overexpressed P450 in a Peruvian population) was functionally characterized. Recombinant CYP6P5 metabolized the type II pyrethroids, deltamethrin and α-cypermethrin with comparable affinities (*K*_M_ of 3.3 μM ± 0.4 and 3.6 μM ± 0.5, respectively), but exhibited a 2.7-fold higher catalytic rate for α-cypermethrin (*k*_cat_ of 6.02 min^−1^ ± 0.2) versus deltamethrin (2.68 min^−1^ ± 0.09). Time-course assays revealed progressive depletion of the above pyrethroids with production of four HPLC-detectable metabolites. Low depletion was obtained with type I pyrethroid, permethrin. Transgenic expression in *Drosophila melanogaster* demonstrated that overexpression of *CYP6P5* alone conferred type II pyrethroid resistance, with only 16% and 55.3% mortalities in flies exposed to 0.25% α-cypermethrin and 0.15% deltamethrin, respectively. Synergist bioassays using P450 inhibitor piperonylbutoxide significantly recovered susceptibility (mortality = 73.6%, *p* < 0.001) in synergized flies exposed to 4% piperonylbutoxide, plus 0.25% α-cypermethrin, compared to non-synergized flies (mortality = 4.9%). Moderate resistance was also observed towards 4% DDT. These findings established the preeminent role of *CYP6P5* in metabolic resistance in *An. albimanus,* highlighting challenges associated with deployment of insecticide-based control tools in the Americas.

## Highlights

• *CYP6P5* is the most overexpressed P450 in *An. albimanus* from Peru.
• *CYP6P5* metabolizes α-cypermethrin and deltamethrin with high catalytic activity.
• *CYP6P5* confers high pyrethroid resistance to transgenic *D. melanogaster*.

## Graphical abstract

**Figure:**
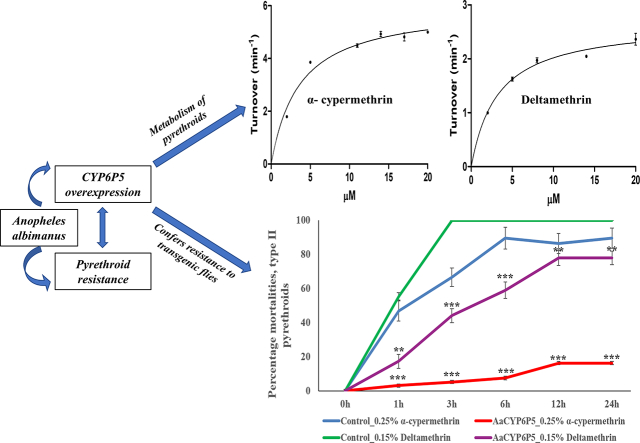

## 1 Introduction

Significant reduction in the global malaria burden was achieved between 2000 and 2015, through widespread deployment of two key vector control tools: long-lasting insecticide-treated bed net (LLINs) and indoor residual spraying (IRS) (Bhatt et al., 2015; WHO, 2020). However, the spread of insecticide resistance is threatening to reverse this progress (Hemingway et al., 2016), with widespread pyrethroid resistance established in the major malaria vectors in Africa (Hancock et al., 2018; Weedall et al., 2019), the Americas (Laporta et al., 2015; Mackenzie-Impoinvil et al., 2019), and Asia (Susanna and Pratiwi, 2022). Progress has been made in understanding the molecular basis of metabolic resistance in some major African malaria vectors like *Anopheles funestus* and *Anopheles gambiae* (Ibrahim et al., 2018; Riveron et al., 2013; Lucas et al., 2019; Miles et al., 2017). Furthermore, recent studies have detected major metabolic resistance markers, e.g. in *An. funestus*, the *GSTe2*-119F (Riveron et al., 2014b), CYP6P9a_R (Weedall et al., 2019) and CYP6P9b_R (Mugenzi et al., 2019), and in *An. gambiae*, the *GSTe2*-119 V (Lucas et al., 2019), which allow for detection of resistance alleles and monitoring of their spread in the field. In contrast, little has been done to elucidate drivers of metabolic resistance and detect important markers in resistance genes in malaria mosquitoes from other regions, such as *A. albimanus*, a major vector in the Americas. Most studies on molecular mechanisms of resistance in *An. albimanus* have focused on target site mechanisms, for example, the knockdown resistance (*kdr*) mutation in the voltage-gated sodium channel (VGSC). These include a report of absence of the *kdr* mutation in segment 6/domain II of the VGSC in population from Columbia (Orjuela et al., 2019), a report of mutations in 1014 codon in populations from Mexico (L1014F), Nicaragua and Costa Rica (L1014C) (Lol et al., 2013), as well as a report on Peruvian populations, in which frequencies of 5% and 15–30% of the L1014S *kdr* mutations was established in deltamethrin- and α-cypermethrin-resistant females (Mackenzie-Impoinvil et al., 2019).

Recently, using genome-wide transcriptional analyses, the P450 *CYP6P5* was shown to be 68 times overexpressed in pyrethroid-resistant *An. albimanus* from Peru, compared to the fully susceptible laboratory colony, Sanarate (Guatemala) (Mackenzie-Impoinvil et al., 2019). However, prior to this study, the functional role of this P450 in pyrethroid metabolism had not been elucidated.

This study aimed to validate the role of the P450, *An. albimanus CYP6P5* in insecticide resistance. The in vitro heterologous expression in *E. coli*, coupled with high-performance liquid chromatography (HPLC), confirmed that the recombinant CYP6P5 metabolize pyrethroids with the production of well-characterised hydroxylated metabolites (Stevenson et al., 2011). Also, the ability of the *CYP6P5* to confer insecticide resistance was validated by overexpressing it in transgenic flies (*Drosophila melanogaster*) followed by insecticide contact bioassays, which confirmed its ability to confer resistance to type II pyrethroids, deltamethrin and α-cypermethrin.

## 2 Materials and methods

### 2.1 Amplification and cloning of full-length *An. albimanus CYP6P5*

The RNA utilized for amplification of *CYP6P5* from cDNA was from field populations described from a previous study (Mackenzie-Impoinvil et al., 2019). This field populations, collected in 2015 (from Puerto Pizzaro, Tumbes, Peru, 3° 30’ 10S, 80° 23′ 38 W) were resistant to deltamethrin and α-cypermethrin. Also, the fully insecticide susceptible, laboratory colony, Sanarate (originally from Guatemala) were used for RNA extraction Total RNA was extracted from three replicates each of 10 females: (i) deltamethrin-resistant, R_d_, (ii) females not exposed to any insecticide (control, of the same age and same population as R_d_), and (iii) Sanarate colony. The RNA was extracted using the Applied Biosystems Arcturus PicoPure RNA isolation kit (Applied Biosystems, CA, USA) according to the manufacturer's instructions. RNA concentration and integrity were assessed using the Agilent 2100 Bioanalyzer (Agilent Technologies, CA, USA). Complementary DNA (cDNA) was synthesized by reverse transcription, from 1 μg of the extracted RNA, using the SuperScript III (Invitrogen, Waltham, CA, USA) with oligo-dT20 and RNAse H (New England Biolabs, Ipswich, MA, USA). The full-length open reading frames of *An. albimanus CYP6P5* (hereafter, *AaCYP6P5*) were amplified from each cDNA separately using the Phusion High-Fidelity DNA Polymerase (Fermentas, Waltham, MA, USA), with forward and reverse primer sets listed in Table S1. Amplification of *AaCYP6P5* was as follows: One microliter of cDNA was added to a total volume of 24 μL premix comprised of 5× Phusion HF Buffer (1.5 mM MgCl_2_ final concentration), 100 μM deoxynucleotide (dNTP) mixes, 0.1 μM each of forward and reverse primers, 0.016 U of Phusion High-Fidelity DNA Polymerase, and 17.55 μL of ddH_2_0. Thermocycling conditions were 98 °C for 5 min; 35 cycles of 98 °C for 30 s (denaturation), 60 °C for 30 s (annealing), extension at 72 °C for 45 s; and a final hold at 72 °C for 10 min (final elongation). The PCR products were gel purified with QIAquick® Gel Extraction Kit (QIAGEN, Hilden, Germany) and ligated into pJET1.2/blunt cloning vector using the CloneJET PCR Cloning Kit (ThermoFisher SCIENTIFIC, Waltham, MA, USA). These were then cloned into the *E. coli, DH5α* and miniprepped., The plasmids were prepared using QIAprep® Spin Miniprep Kit (QIAGEN, Hilden, Germany) and sequenced on both strands using the primers mentioned above.

### 2.2 Genetic variability of *An. albimanus CYP6P5*

To identify genetic variants in *An. albimanus CYP6P5* from Peru, data from a differential gene expression analysis (NCBI BioProject PRJNA49810) (Mackenzie-Impoinvil et al., 2019) was re-analyzed. In that experiment, three replicates each of mosquitoes resistant to deltamethrin, α-cypermethrin and unexposed (control), collected in Puerto Pizarro, Tumbes, Peru in October 2015 were compared with the susceptible Sanarate (Guatemala) colony of *An. albimanus*. Here, data from replicates for each treatment were combined into a single sample per treatment. A total of 30 haploid genomes were included per treatment as each replicate in (Mackenzie-Impoinvil et al., 2019) consisted of five mosquitoes. Reads were aligned to the *An. albimanus* STECLA genome (VectorBase v54) with BWA, v0.7.17 (Li, 2013), sorted with picard tools, v2.26.4 (https://broadinstitute.github.io/picard/), and variants called with freebayes, v1.3.5 (Garrison and Marth, 2012) for the *CYP6P5* gene, and a flanking region of 1000 bp. Freebayes predicted variants were filtered to remove indels and keep biallelic variants with a minimum phred-scaled quality of 20, using vcflib v1.0.2 (Garrison et al., 2021). Remaining variants were annotated using SnpEff v5.0 (Cingolani et al., 2012) and used to create a lollipop plot in the R package ggplot2 (Wickham, 2011) of variants with a frequency greater than 5% across the gene-body of *CYP6P5* for each of the four treatments included. As established by (Mackenzie-Impoinvil et al., 2019), *CYP6P5* was over-expressed in the Peru population versus Sanarate, meaning variant prediction was based on unbalanced read-depths (File S1, for examples from missense mutations). This was offset by the intention to identify variants prevalent in the Peru population.

### 2.3 Sequence characterization and in silico prediction of insecticide-metabolizing activity of *An. albimanus CYP6P5*

The amino acid coding sequence of *CYP6P5*(sequencing from section 2.1) was mapped to the sequence of *Pseudomonas putida CYP101A* (P450cam) (Gotoh, 1992b; Poulos et al., 1985), to establish putative substrate recognition sites 1 to 6 (SRS 1–6), compared with the sequences from *An. funestus CYP6P5*, *An. gambiae CYP6P5* and the human *CYP3A4* (Yano et al., 2004).

The homology model and energy minimization of CYP6P5 sequences was built using MODELLER 10.1 (Sali and Blundell, 1993) with the crystal structure of human *CYP3A4* (PDB ID: 1TQN, ~34.8% similarity, 1e^−75^) (Yano et al., 2004) as a template. A total of 20 models were generated for the predominant sequence of *CYP6P5* and assessed externally using Errat (version 2), to identify the best model from statistical patterns of non-bonded interaction between different atom types (Colovos and Yeates, 1993). Overall quality scores were 53.36% for the highest ranked model. The virtual ligand structures for α-cypermethrin (PubChem ID:93357) were downloaded from PubChem database (https://pubchem.ncbi.nlm.nih.gov/compound/101618973), while deltamethrin (ZINC01997854), 1*R*-*cis* permethrin (ZINC01850374) and DDT (ZINC01530011) were retrieved from the library in ZINC^12^ database (https://zinc.docking.org/) (Irwin and Shoichet, 2005). Preparation of the receptor and ligand and docking were carried out using the Molegro Virtual Docker version 7.0 (Thomsen and Christensen, 2006). Five cavities were detected for the CYP6P5 model, with the biggest having a volume of 1880.06 Å. Docking was carried out with MolDock scoring function, with the above cavity as a constraint, targeting a binding site of 20 Å radius above the heme iron, and pose clustering of 12 solutions.

### 2.4 In vitro characterization of metabolic activity of *AaCYP6P5*

#### 2.4.1 Cloning of recombinant AaCYP6P5 and heterologous expression in *E. coli*

The *AaCYP6P5* cDNA of the predominant allele from the Peru samples (sequenced in section 2.1) was prepared for expression using a previously described protocol (Pritchard et al., 1997) by fusing bacterial ompA+2 leader sequences to the 5′ of CYP6P5, using primers provided in Table S1. The PCR products were cleaned, digested with restriction enzymes *Nde*I and *Xba*I and ligated into the expression vector pCWori+ already linearized with the same restriction enzymes, to create a construct pB13::ompA+2-AaCYP6P5. This recombinant plasmid was co-transformed together with *An. gambiae* CYP450 reductase (pACYC-AgCPR) into *E. coli JM109* (Pritchard et al., 1997). The recombinant AaCYP6P5 was expressed optimally at 21.5 °C, with shaking at 150 rpm for 72 h, following induction with 0.5 mM δ-aminolevulinic acid (δ-ALA) and 1 mM isopropyl β-D-1-thiogalactopyranoside (IPTG). The P450 content in the membrane was determined spectrophotometrically (Omura and Sato, 1964) and cytochrome P450 reductase activity was determined using the cytochrome *c* reduction assay (Guengerich et al., 2009).

#### 2.4.2 Pyrethroid metabolic assays

The ability of recombinant AaCYP6P5 to metabolize pyrethroids was investigated using substrate depletion assays with permethrin, deltamethrin and α-cypermethrin. The protocols for incubation and high-performance liquid chromatography (HPLC) analyses for the above insecticides followed procedures previously published (Ibrahim et al., 2018) with modifications. The depletion assay mix contained 0.225 μM membrane expressing recombinant AaCYP6P5, 1.8 μM cytochrome *b*_5_ (originally from *An. gambiae*), 20 μM insecticide, diluted to 100 μL with water. The reaction was started by adding 100 μL of 1 mM final concentration of either NADPH or NADPH regeneration buffer. The NADPH regeneration buffer contained 1 mM glucose-6-phosphate, 0.1 mM NADP^+^, 0.25 mM MgCl_2_ and 1 unit/mL glucose-6-phosphate dehydrogenase. The negative reactions contained the same regeneration buffer mix but without the NADP^+^ (equal volume of buffer added in place of the amount of the buffer containing NADP^+^). The depletion assay with NADPH was carried out using NADPH dissolved in 50 mM phosphate buffer, pH 7.4. The negative reactions contained water alone, added to 50 mM phosphate buffer at pH 7.4, without NADPH. Reactions were conducted in triplicate for positive and negative incubations, for each insecticide. Following incubation for 2 h at 30 °C and 1200 rpm, reactions were quenched with 200 μL of ice-cold acetonitrile and samples were incubated for an additional 10 min to dissolve the insecticides, before centrifugation at 20,000 *g* for 20 min. The supernatants (150 μL each) were loaded into the HPLC vials for analysis. The quantity of insecticide remaining in the samples was determined by reverse-phase HPLC, with UV absorbance of 226 nm, using a C18 column (Acclaim 120™, Dionex). From the vials, 100 μL of sample was loaded with a flow rate of 1 mL/min at 23 °C into an isocratic mobile phase of 80% acetonitrile and 20% water. The retention times for deltamethrin, α-cypermethrin and permethrin were 10.19 min, 9.78 min, and 11.21 min (*trans*), 12.5 min (*cis*), respectively. A paired *t*-test was used to compare positive reactions (+NADPH) vs negative reactions (-NADPH) for the depletion of the substrates.

Time course depletion assays of the insecticides were carried out in 200 μL reaction mixes containing 20 μM of either deltamethrin or α-cypermethrin, 0.1 μM AaCYP6P5 membrane, 0.8 μM cytochrome *b*_5_, and NADPH. Incubation conditions were as above between, 0–120 min.

Steady state kinetic parameters were determined with α-cypermethrin and deltamethrin, by measuring the rate of reaction for 1 h while varying the substrate concentrations (0–20 μM) in the presence of NADPH, 0.1 μM AaCYP6P5 membrane and 0.8 μM cytochrome *b*_5_. Reactions were performed in triplicate both for +NADPH and –NADPH, for each concentration. Values of *K*_M_ and V_max_ were calculated from the Michaelis-Menten plot using the least squares, non-linear regression in the GraphPad Prism 5.0 (GraphPad Inc., La Jolla, CA, USA).

### 2.5 In vivo characterization of the role of *AaCYP6P5* in resistance

#### 2.5.1 Transgenic expression of *AaCYP6P5*

To confirm if over-expression of *CYP6P5* alone can confer resistance to the pyrethroid insecticides, transgenic flies expressing this gene were generated using GAL4/UAS system. The preparation of the transgenic flies followed the protocols described previously (Riveron et al., 2013). The full-length CYP6P5 was amplified from the predominant allele used for in vitro analyses, with Phusion High-Fidelity DNA Polymerase (Thermo Fisher Scientific, MA, USA) using primers bearing *Bgl*II and *Xba*I restriction sites (Table S1). The PCR products were cleaned and cloned into the pUASattB vector linearised with the above restriction enzymes. Using the PhiC31 system, clones were injected into the germ-line of a *D. melanogaster* line carrying the attP40 docking site, 25C6 on chromosome 2 [*y w M(eGFP, vas-int, dmRFP)ZH-2A; P{CaryP}attP40*]. Microinjection and balancing of UAS stock to remove integrase was carried out by Fly Facility (Cambridge, UK) generating a UAS-CYP6P5 transgenic line. Ubiquitous expression of the transgene in adult F_1_ progeny (experimental group) was obtained after crossing virgin females from the GAL4-Actin driver strain Act5C-GAL4, BL25374 [y[1] w[*]; P{Act5C-GAL4-w}E1/CyO, 1;2] (Bloomington, IN, USA) with the UAS-CYP6P5 males. Similarly, adult F_1_ control progeny (control group) with the same genetic background as the experimental group but without the *CYP6P5* insert were obtained by crossing virgin females from the driver strain Act5C-GAL4 and UAS recipient line males with white eyes (not carrying the pUASattB-CYP6P5 insertion).

#### 2.5.2 Drosophila contact bioassays

For the contact bioassay, 2 to 4-day old experimental and control F_1_ females were exposed to 0.25% α-cypermethrin (5× the recommended dose of 0.05% for *Anopheles* mosquitoes), 0.15% deltamethrin, 2% permethrin and 4% DDT-impregnated papers, prepared in acetone and Dow Corning 556 Silicone Fluid (BHD/Merck, Hesse, Germany). These papers were rolled and introduced into 45 cc plastic vials to cover the entire wall. The vials were plugged with cotton soaked in 10% sucrose. Minimum of 25 flies were placed in each vial, and the mortality plus knockdown was scored after 1 h, 3 h, 6 h, 12 h and 24 h. For each insecticide, assays were performed in four replicates and Student's *t*-test used to compare the mortalities between the experimental groups and the control. A synergist bioassay was also conducted using four replicates of 25 females, which were exposed to 4% PBO for 1 h, followed by exposure to papers impregnated with 0.25% α-cypermethrin.

To confirm overexpression of *CYP6P5* in the experimental group three replicates each of 6 F_1_ females were used for qRT-PCR, using a previously established protocol (Riveron et al., 2014a). Total RNA was extracted, and cDNA was synthesized as described above, and the relative expression levels of the transgene were assessed using the experimental F_1_ progeny as well as two controls [(i) parental, white-eye flies with no *CYP6P5* insertion, crossed with GAL4/UAS driver lines, and (ii) parental, red-eyed, UAS-CYP6P5 flies not crossed with GAL4/UAS lines], with normalization using the *RPL11* housekeeping gene. The primers used are provided in Table S1.

## 3 Results

### 3.1 DNA and amino acid sequences characterization of *AaCYP6P5*

**Fig. 1:**
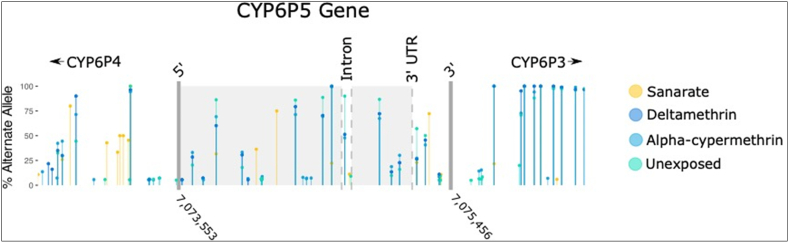
**Lollipop chart of the frequency of alternate alleles across the *CYP6P5,* between mosquitoes from Peru (shades of blue) and the susceptible Sanarate strain (Orange).** Shades of blue were chosen as the frequency of variants in Peru was similar across treatments. Y-axis is the frequency expressed as a percentage of the non-reference allele with frequency > 5% for all predicted bi-allelic sites. The x-axis is the chromosomal location of *CYP6P5* with 5′ start and 3′ end labeled by position and thick grey bars on chromosome 2R of the *An. albimanus* genome. The light grey shaded region delineates coding sequence within the gene and dashed grey lines the location of the single intron and start of the 3’ UTR. The 5’UTR is not shown as it is very short (27 bp). (For interpretation of the references to colour in this figure legend, the reader is referred to the web version of this article.)

Analysis of the polymorphism patterns of the *CYP6P5* gene and 1 kb flanking regions (total 3.8 kb) in RNASeq data from the Sanarate and field pyrethroid resistant population from Peru, revealed moderate polymorphism. There were 22 coding sequence variants, including 12 non-synonymous variants, and two intronic variants. When plotted by frequency across the gene (lollipop chart, Fig. 1), higher-frequency variants occurred together across deltamethrin, α-cypermethrin and unexposed treatments, and more commonly, than in Sanarate, which is not surprising given the close proximity of the STECLA reference sequence (from El Salvador) and the susceptible Sanarate strain (from Guatemala). Two of the twelve missense mutations occurred at high frequency in Peru (71–86% and 67–87%, File S1), the first changes asparagine to histidine at amino acid 264 (N264H) and the second lysine to asparagine at position 437 (K437N).

**Fig. S1:**
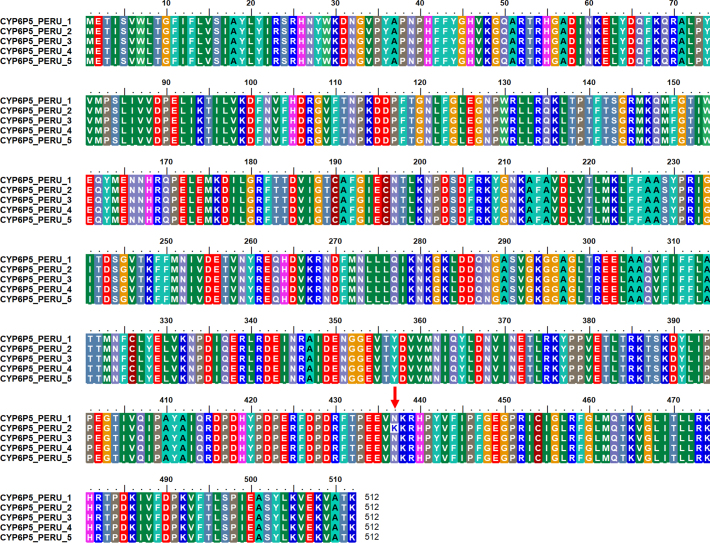
Alignment of *AaCYP6P5* from the deltamethrin resistant samples from Peru showing two alleles differentiated by a K437N mutation.

**Fig. S2:**
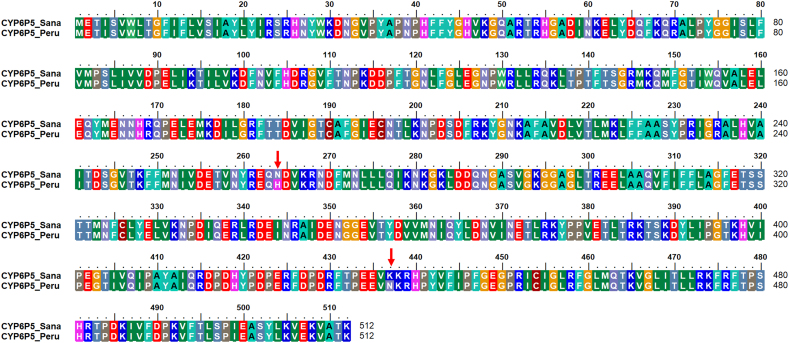
Alignment of *AaCYP6P5* from the deltamethrin resistant Peru samples and the manually trimmed Sanarate nucleotides translated to their respective amino acid sequences showing the two mutations N265H and K437N.

Five of the Sanarate clones sequenced had introns. *AaCYP6P5*, located on the chromosome 2R, has two exons and one intron, and only one splice variant. Two of the Sanarate clones, manually spliced and aligned to the full-length *AaCYP6P5* had nucleotide insertions that distorted the open reading frame, causing protein truncation when translated. The alignment of the amino acid sequences of the cloned *AaCYP6P5* samples from Peru showed four of the sequences had a mutation at position 437 (K437N), as above for the RNASeq data (Fig. S1). The sequence of this dominant allele has been deposited in GenBank (Accession number MW629015). After removing the intron, the manually trimmed Sanarate sequences were translated and aligned with the sequences from Peru (Fig. S2). This alignment revealed only one amino acid change, same as that of the variant allele with the K437N mutation observed in the Peru sample (Fig. S1).

**Fig. S3:**
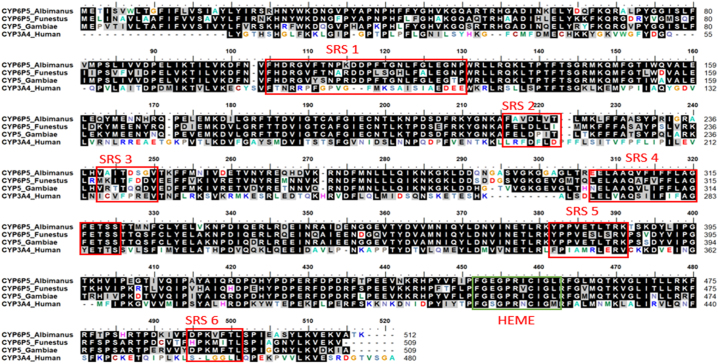
Sequence alignment of *AaCYP6P5, AfCYP6P5* and *AgCYP6P5*, along with the sequence of structurally resolved human *CYP3A4* (PDB ID: 1TQN).

The amino acid sequences of *AaCYP6P5* were compared with those of *An. gambiae* and *An. funestus*, the two major African malaria vectors (Fig. S3). The six conserved substrate recognition sites (SRS) of the P450s showed more mutational changes in the *An. albimanus* amino acid sequence in SRS-2 and -3 compared to those of *An. gambiae* and *An. funestus* (Fig. S3). A percentage identity matrix revealed that *An. albimanus CYP6P5* is 76.03% and 74.80% identical to *An. gambiae* and *An. funestus*, respectively.

### 3.2 Prediction of insecticide-metabolizing ability of *AaCYP6P5* using molecular docking simulation

**Fig. 2:**
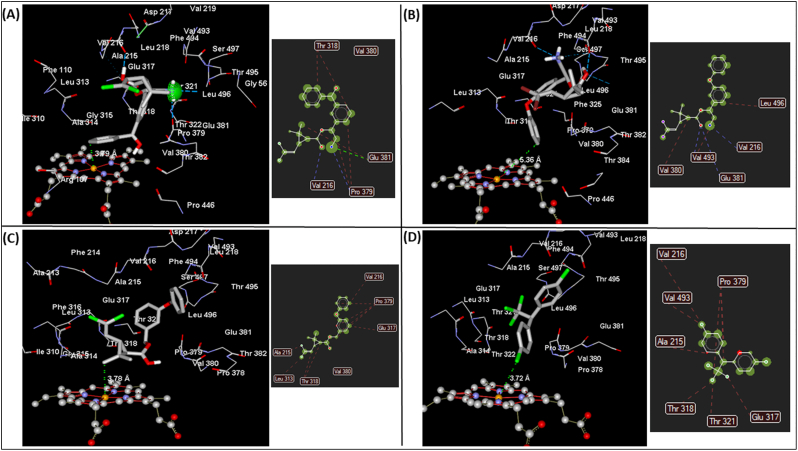
**Predicted binding mode and intermolecular interactions of insecticides in the active site of *An. albimanus* model. (A)** α-cypermethrin, (B) deltamethrin, (C) permethrin and (D) DDT. Insecticides are in stick format and intermolecular interactions are presented in dashed lines, and as inset in each of the four panels. Distances between possible sites of metabolism and the heme iron are annotated in Angstrom.

Molecular docking predicted metabolism of the pyrethroid insecticides, particularly type II class. The α-cypermethrin docked (1st ranked pose, Table S2) productively into the active site of CYP6P5 model, with the 4′ spot of phenoxy ring oriented at 3.79 Å from the heme iron (Fig. 2A), suggesting ring hydroxylation to produce 4′-hydroxy-α-cypermethrin. The 4′-hydroxy is known to be a major route of pyrethroid metabolism in recombinant *An. gambiae* CYP6M2 (Stevenson et al., 2011), and other organisms (Gilbert and Gill, 2010). Intermolecular interactions include two hydrogen bonds (Fig. 2A, inset): (i) donated by the α-cyano nitrogen atom to SRS-5 Pro^379^ (2.97 Å distance and − 1.07 kcal/mol), and (ii) donated by the ester oxygen (2.58 Å distance and − 2.36 kcal/mol) to Val^216^ from SRS-2. Electrostatic interaction was also predicted between the α-cyano group and Glu^381^, as well as steric interactions with Thr^318^, Val^380^, Glu^381^ and Pro^379^.

The 1st ranked pose of deltamethrin also docked productively but with a lower score than α-cypermethrin Fig. 2B, Table S2). The C-4′ spot of phenoxy ring was oriented above the heme, at 5.36 Å, suggesting ring hydroxylation to 4′-hydroxy-deltamethrin as the major metabolic route. Intermolecular interactions include three hydrogen bonds (Fig. 2B, inset): (i) donated by the α-cyano nitrogen atom to Val^216^ (2.50 Å and − 1.64 kcal/mol), (ii) donated by the ester oxygen (2.59 Å and − 2.49 kcal/mol) to Val^493^, and (iii) the ester oxygen to Glu^381^ (2.97 Å and − 1.19 kcal/mol); as well as electrostatic interaction with Val^380^, Glu^381^, Val^493^, Val^216^ and Leu^496^.

Permethrin docked with the cis−/*trans*-methyl group of cycloprane ring oriented above the heme at 3.78 Å (Fig. 2C) suggesting cis−/*trans*-methyl hydroxy metabolite, known to be a minor pathway for pyrethroid metabolism (Stevenson et al., 2011; Gilbert and Gill, 2010). Neither hydrogen bonding, nor electrostatic interactions contributed to the binding energetics of permethrin (Fig. 2C, inset). However, an extensive network of steric interactions was observed between permethrin and Ala^215^ and Val^216^ of SRS-2, Leu^313^, Glu^317^, Thr^318^, Val^380^ and Pro^379^.

For DDT (which exhibited the lowest MolDock Score), binding in the first 3 top ranked poses were unproductive. The C-1 of the trichloroethyl group is 9.4 Å from the heme iron, in the top ranked pose (Fig. 2D). One of the chlorine atoms of the dichlorophenyl rings is projected above the heme, at 3.72 Å. As was the case with permethrin, neither hydrogen bonds nor electrostatic interactions were observed in this pose (Fig. 2D, inset) but a network of steric interactions was seen with Ala^215^, Val^216^, Val^493^, Thr^318^, Thr^321^, Glu^317^ and Pro^379^.

### 3.3 Validation of recombinant AaCYP6P5 metabolism of pyrethroids

#### 3.3.1 Substrate depletion assays

**Fig. 3:**
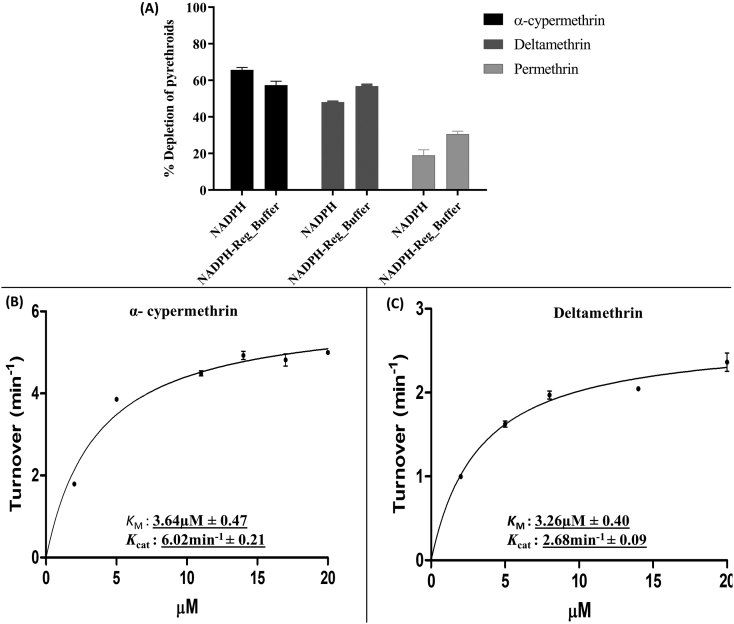
**AaCYP6P5 metabolism of type II pyrethroids. (A)** Percentage depletion of type I and II pyrethroids from *AaCYP6P5*-mediated metabolism**.** Each insecticide assays were carried out independently with both 1 mM of NADPH and NADPH regenerating system. **(B and C)** Michaelis–Menten plot of AaCYP6P5 metabolism of α-cypermethrin and deltamethrin. Inserts are the Michaelis constant (*K*_M_) and turnover (catalytic constant, *K*_cat_).

**Fig. S4:**
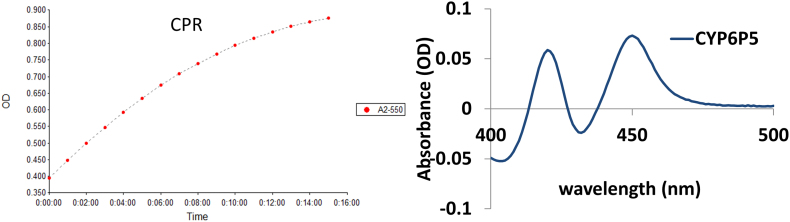
Measurement of the CPR and P450 contents of the expressed membrane. (A) *Anopheles gambiae* cytochrome P450 reductase co-expressed with *An. albimanus.* (B) Fe^2+^ -CO vs. Fe^2+^ difference spectrum of membrane expressing *An. albimanus* CYP6P5 from Peru. The yield of the CPR was 0.95 nmol/min/μL and the P450, 8.5 μM - 15 μM purified membranes.

The recombinant AaCYP6P5 expressed with concentration of15 μM of purified membrane, and with cytochrome P450 reductase activity of 285 nmol of cytochrome *c* reduced per min/mg protein (Figs. S4). To compare the efficiency of electron transfer to recombinant CYP6P5, metabolic assays were conducted with pyrethroids using both the NADPH regeneration system and NADPH alone. The AaCYP6P5 metabolised permethrin moderately with 30.6% ± 2.8 depletion when electrons were supplied from the NADPH regenerating system (*p* < 0.05 versus NADP- incubation), and 21.8% ± 5.3 (p < 0.05) when using 1 mM NADPH (Fig. 3A). It metabolised the type II pyrethroids α-cypermethrin and deltamethrin with depletion of 57.4% ± 3.8 (*p* < 0.01) and 56.8% ±1.3 (p < 0.01), respectively, when the NADPH regenerating system was used and 65.7% ± 2.4 (p < 0.01) and 48.1% ± 0.3 (p < 0.01) respectively, when electrons were supplied by NADPH.

#### 3.3.2 Estimation of steady-state kinetic parameters and identification of HPLC metabolites

The steady state kinetics for depletion of type II pyrethroids followed Michaelis-Menten pattern (Fig. 2B and -C). Recombinant CYP6P5 had comparable affinity for α-cypermethrin and deltamethrin, with *K*_*M*_ of 3.64 μM ± 0.47 and 3.26 μM ± 0.40, respectively. However, it had 2.7-fold higher turnover for α-cypermethrin compared to deltamethrin, with a maximum *k*_cat_ of 6.02 min^−1^ ± 0.21 and 2.68 min^−1^ ± 0.09, respectively. This resulted in catalytic efficiencies of 1.65 min^−1^ μM^−1^ and 0.82 min^−1^ μM^−1^ for α-cypermethrin and deltamethrin, respectively.

**Fig. 4:**
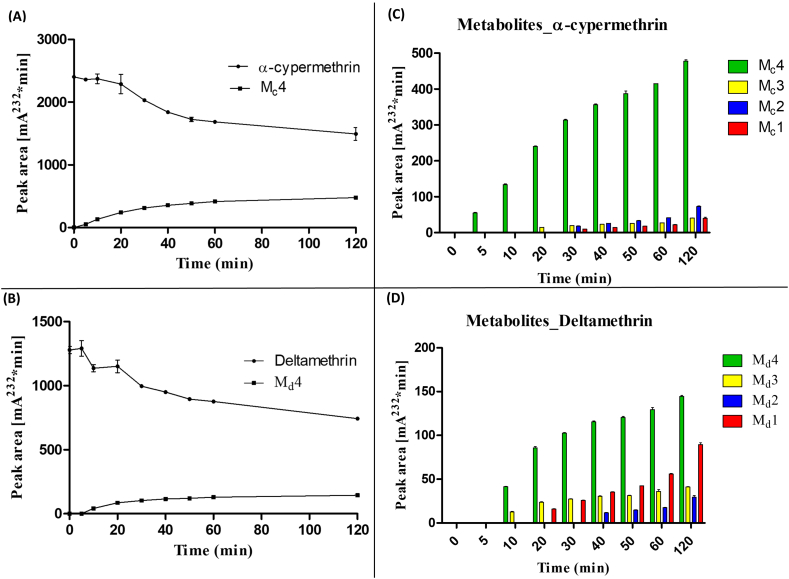
**Time course assays of depletion of α-cypermethrin and deltamethrin.** (**A** and **B**) Depletion of parent compounds and production of the major metabolite M1. (**C** and **D**) Relative production of metabolites M_d_1 – M_d_4.

**Fig. S5:**
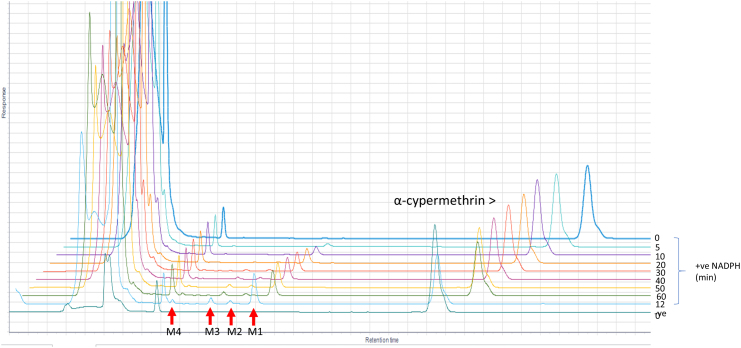
HPLC chromatograms (off-set) of the time-course of α-cypermethrin showing formation of metabolites (0-120 min). P450 = 0.225 μM; insecticide = 20 μM.

**Fig. S6:**
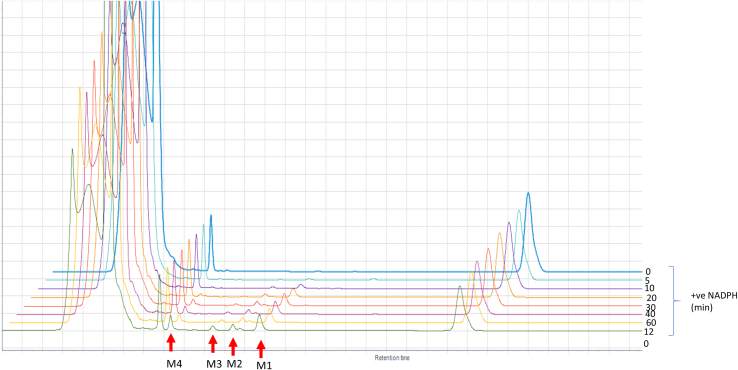
HPLC chromatograms (off-set) of the time-course of deltamethrin with metabolites showing formation of metabolites (0-120 min). P450 = 0.225 μM; insecticide = 20 μM.

**Fig. S7:**
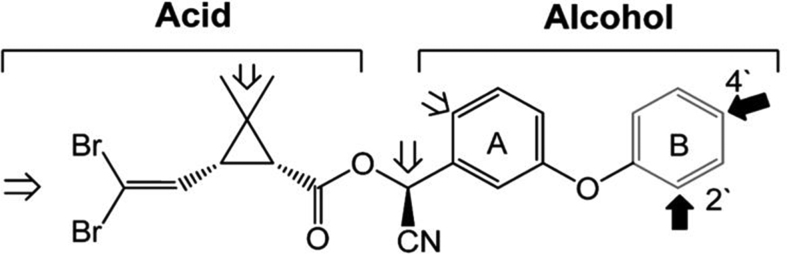
Structure of deltamethrin with constituent acid and alcohol moieties. Primary sites of P450 hydroxylation are indicated by bold arrows at the 2′ and 4′ positions, and minor routes of hydroxylation are indicated with open arrows.

Time-course assays revealed progressive production of four metabolites from α-cypermethrin and deltamethrin (Fig. S5, Fig. S6). The pattern of the retention times for these metabolites are similar for both insecticides (Table S3). However, the higher turnover rate of the metabolism of α-cypermethrin compared to deltamethrin was also reflected by depletion starting earlier with α-cypermethrin (Fig. 4A). The major and most abundant metabolite (M_c_4) appeared within 5 min of incubation for α-cypermethrin, with M_c_3 produced 20 min into the reaction, while M_c_2 and M_c_1 both appeared 30 min into the reaction. However, the metabolic reaction did not start until after 10 min for deltamethrin, with the production of M_d_4 and M_d_3 metabolites. Interestingly, M_d_1 appeared 20 min earlier for deltamethrin metabolism (at 20 min) compared to M_d_2 which appeared at 40 min. These are hydroxylated metabolites previously described for the recombinant CYP6M2 from *An. gambiae* (Stevenson et al., 2011). The primary sites of P450 hydroxylation of pyrethroids are shown in Fig. S7 with bold arrows at the 2′ and 4′ positions, while the minor routes of hydroxylation are shown with open arrows (Khambay and Jewess, 2004).

### 3.4 Validation of the role of *AaCYP6P5* in pyrethroid resistance using transgenic flies

**Fig. 5:**
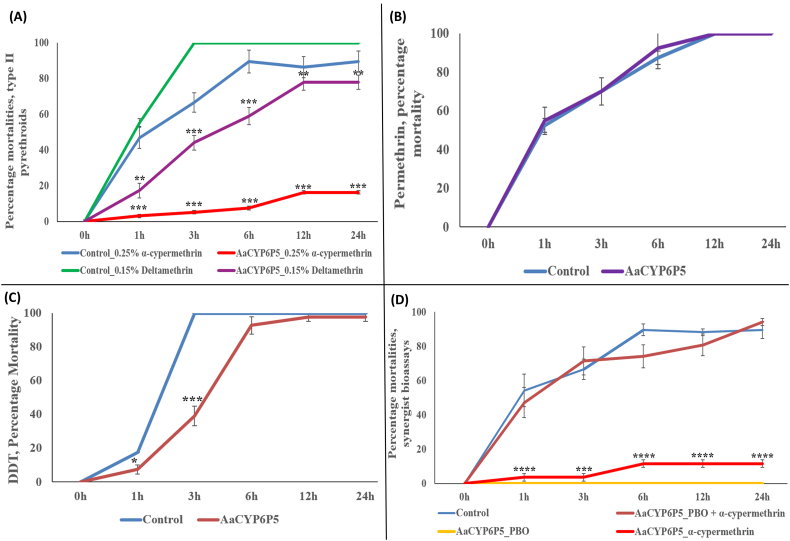
**Susceptibility bioassays with transgenic flies**. **a:** Progeny of crosses between Actin5C-GAL4 and UAS-AaCYP6P5 (transgenic flies over-expressing *AaCYP6P5*) with 0.05% and 0.25% α-cypermethrin vs. control flies; b**:** with 0.15% deltamethrin; c**:** with 4% DDT; and d**:** results of synergist bioassays with pre-exposure to 4% PBO followed by 0.05% α-cypermethrin. Each point is a mean ± S.E.M. of four independent replicates comprise of 20–25 flies. Significantly different: *p < 0.05, ** *p* < 0.01, *** *p* < 0.001, **** *p* < 0.0001.

Contact bioassays carried out using 0.25% (5× the discriminating dose for the *Anopheles* mosquitoes) α-cypermethrin revealed a significantly high resistance in the experimental flies (transgenic Act5C-CYP6P5 females) compared to control flies, at all five different times, spanning 1 h – 24 h (mean mortality = 9.7% in Act5C-CYP6P5 flies vs 75.8% in control flies, *p* < 0.001) (Fig. 5A). Specifically, at 1 h, mortality in the experimental flies was 3.1% compared to 46.9% for control flies (p < 0.001). These mortalities increased for the experimental flies to 5.2%, 7.5%, 16.3% and 16.3% at 3 h, 6 h, 12 h and 24 h, respectively, compared with the control flies which exhibited mortalities of 66.5%, 89.5%, 86.4% and 89.5% for the respective times (*p* < 0.001).

Significant differences were also observed from exposure to 0.15% deltamethrin (Fig. 5A), with transgenic flies over-expressing *CYP6P5* exhibiting mortality of only 17.3% at 1 h compared with 55.3% for control flies (*p* < 0.01). Mortalities increased to 44.1%, 59%, 78% and 78% at 3 h, 6 h, 12 h and 24 h for the experimental flies compared with 100% mortalities obtained from control flies, for the rest of the times. Mean mortality for the experimental flies was 55.3% compared to 91.1% for the control flies (*p* < 0.001).

No resistance was observed when experimental flies were exposed to 2% permethrin. The mortalities obtained were similar between the experimental and the control flies, with average mortalities of 82% and 83%, respectively (Fig. 5B). The mortalities increased linearly from ~55% for both experimental and control flies at 1 h, to 87.5% and 92.5% by 3 h, reaching 100% by the 12th hour.

Differences were also seen in mortalities from exposure to 4% DDT (Fig. 5C), between the experimental flies and the controls, but only at 1 h (mortalities of 7.4% vs 17.5%, *p* < 0.05) and 3 h (39.1% vs 100%, p < 0.001), with overall mean mortality of 66.9% in experimental flies, compared to 83.5% for the control flies (p < 0.05).

Taken together, these results confirmed that over-expression of *AaCYP6P5* alone is sufficient to confer resistance to type II pyrethroids (deltamethrin and α-cypermethrin), and to possibly DDT.

To further validate the role of *AaCYP6P5* in α-cypermethrin resistance a bioassay was repeated with pre-exposure to 4% PBO, followed by 0.25% α-cypermethrin. A significant recovery of susceptibility was observed in the flies pre-exposed to the PBO with mortalities increasing to 47.2% at the first 1 h compared to 3.8% in the repeated conventional assay with α-cypermethrin only (p < 0.001) (Fig. 5D). The mortalities in the PBO-synergized flies rapidly increased to 93% at 3 h compared to no change (3.8%) with the flies exposed to the α-cypermethrin alone (*p* < 0.0001). A moderate increase in mortality (11.7%) in the flies with α-cypermethrin exposure only was seen at 6 h, 12 h and 24 h, while the mortality in the PBO-synergised flies increased respectively to 95%, 97% and 93%. All in all, mean mortality for synergised flies was 73.6%, compared to 4.9% in the flies exposed to α-cypermethrin alone (p < 0.001). No mortality was obtained in flies exposed to PBO only.

The qRT-PCR established the overexpression of the *AaCYP6P5* with a fold change (FC) of 598.4 ± 26.95 in the GAL4-UAS-CYP6P5 flies overexpressing the P450, compared with control flies (control I, progenies of crosses between the parental line flies with no *CYP6P5* insertion, crossed with GAL4/UAS driver line). Also, a FC of 488.6 ± 17.22 was obtained from the GAL4-UAS-CYP6P5 flies compared with the second set of control flies (control II, parental UAS-CYP6P5 flies with red eyes, not crossed with GAL4/UAS driver lines).

## 4 Discussion

The overexpression of *CYP6P5* in field caught *An. albimanus* from Peru has previously been linked to pyrethroid resistance (Mackenzie-Impoinvil et al., 2019). In this study, we functionally validated *AaCYP6P5* as a pyrethroid metabolizing P450, able to metabolize type I and II pyrethroids with high efficiency for the type II; and confer type II pyrethroid resistance in transgenic *D. melanogaster*. Initial reanalysis of RNAseq data and sequencing of CYP6P5 cDNA revealed moderate polymorphism, with no signature of selective sweep, though some non-synonymous mutations were observed when sequences of field population were compared with those of the Sanarate.

The recombinant AaCYP6P5 metabolizes α-cypermethrin and deltamethrin with a high depletion but has low activity for permethrin. Its ortholog from *An. gambiae* (*AgCYP6P5*), metabolizes permethrin with 56.8% depletion, but was shown to deplete lower quantities of deltamethrin (47%) (Yunta et al., 2019). The dissimilarity in amino acid composition among these orthologs is observed in all six SRSs. P450s have six conserved substrate recognition sites (SRSs) (Gotoh, 1992a), with SRS 1, SRS 4, SRS 5 and SRS 6 involved in the P450 catalytic site activities, while SRS 2 and SRS 3 are involved in the substrate access and channelling (Schuler and Berenbaum, 2013). The amino acid sequence of *AaCYP6P5* is more closely related to that of *AgCYP6P5* than to *An. funestus CYP6P5* (*AfCYP6P5*) in SRSs 1, 4, 5 and 6. Since those are the SRSs important for catalysis, this could explain why *AaCYP6P5* and *AgCYP6P5* depleted higher amount of pyrethroids, compared with the *AfCYP6P5* (data on this P450 not yet published).

The amount of pyrethroid turned over by the recombinant AaCYP6P5 is higher than observed from AgCYP6M2 and AgCYP6P3, the two well characterised P450s linked with insecticide resistance in *An. gambiae*. The documented *k*_cat_ for AgCYP6M2 (Stevenson et al., 2011) and AgCYP6P3 (Müller et al., 2008) are 1.2 min^−1^ and 1.8 min^−1^, respectively for deltamethrin, which are lower than the 2.7 min^−1^ for AaCYP6P5*.* The specificity constant, which is the measure of the catalytic performance of an enzyme, showed AaCYP6P5 to be 1.4-fold more efficient than AgCYP6M2 and 2.7-fold more efficient than AgCYP6P3 at metabolizing deltamethrin*.* The metabolites generated in the deltamethrin time-course assay with recombinant AaCYP6P5 further established similarity in depletion of deltamethrin with AgCYP6M2, which was extensively studied using the LC-MS/MS and NMR to reveal points of hydroxylation (Stevenson et al., 2011). The four metabolites identified in the deltamethrin depletion of AgCYP6M2*,* arranged in ascending order of hydrophilic property and retention times (RT) on the HPLC, are 4′-hydroxydeltamethrin; *trans*-hydroxymethyl-deltamethrin; cyano (3-hydroxyphenyl)methyl deltamethrate; and deltamethric acid (Stevenson et al., 2011). The chromatograms obtained from these metabolites are similar to those produced by AaCYP6P5. The first two metabolites to appear are hydroxylation reactions and are the primary metabolites. The last two are secondary metabolites from 4′ hydroxydeltamethrin (Stevenson et al., 2011). We did not observe reduction in the rate of the first metabolite (M4) of AaCYP6P5. However, the higher efficiency of over 2-fold metabolism of deltamethrin from AaCYP6P5, compared with AgCYP6M2 and the observed 2-fold rate of first step of depletion producing M4 (which was reduced to 1-fold once the other metabolites appeared 5 min after) indicated continuous production of M4, while some of it are being broken down to produce the secondary metabolites (as established for AgCYP6M2).

The higher α-cypermethrin metabolism efficiency byAaCYP6P5 (compared with deltamethrin) is probably due to the high level of α-cypermethrin resistance detected in *An. albimanus* from Peru. Only 20% mortality was recorded following exposure to α-cypermethrin, in contrast to 80% mortality from deltamethrin bioassays (Mackenzie-Impoinvil et al., 2019). This is not surprising since α-cypermethrin has been a major pyrethroid component in pesticides commonly used in agriculture in Peru (Lange, 2006), and the potential contamination of the larval habitats of malaria vectors (Quiñones et al., 2015) may result in the routine exposure of *An. albimanus* to this insecticide, accelerating selection pressure.

In the recombinant protein metabolic assays, contrasting differences were observed in the percentage depletion of pyrethroids when electrons were supplied by the NADPH-regeneration buffer or the NADPH directly (with higher depletion of deltamethrin, compared with α-cypermethrin when regeneration buffer was used). However, these minor differences were not significant and suggest that either the regeneration buffer or the NADPH can be used as a source of electron for metabolic assays.

This present study has validated the overexpression of *CYP6P5* from Peruvian *An. albimanus* as a principal detoxification enzyme causing resistance to α-cypermethrin, deltamethrin, and potentially DDT. Though recombinant enzyme metabolic assays have shown this P450 to moderately metabolize permethrin, transgenic analysis using *D. melanogaster*, as well as molecular docking indicated that it does not confer resistance to this type I pyrethroid. However, it may possibly contribute cross resistance to other insecticides not characterised in this study. Indeed, it's ortholog, *AgCYP6P5* has been confirmed to metabolize other insecticides, including the juvenile hormone analogue, pyriproxyfen (Yunta et al., 2019).

## 5 Conclusion

This study has demonstrated and functionally validated *AaCYP6P5* as an important P450 which metabolizes key insecticides used in malaria vector control, enhancing our understanding of metabolic resistance in *An. albimanus*, a major malaria vector from the Americas. However, both in vitro and in vivo characterization utilized indicated that this P450 specializes in metabolism of the type II pyrethroids, suggesting the contrasting pattern of metabolic resistance. Further characterization of this P450, for example, analysis of the role of the mutations observed in its coding sequences and characterization of its 5′ regulatory elements is recommended. These may lead to identification of genetic markers linked with its overactivity, which can be used to create tests for the diagnostic markers, in the future. Such information will be important for vector control programmes and stakeholders for management of resistance in the *An. albimanus*.

The following are the supplementary data related to this article.

Supplementary material 1 Image 1

Supplementary material 2 Image 2

## Availability of data

DNA sequences reported in this paper were deposited at GenBank (Accession No. MW629015). RNASeq data were deposited under NCBI BioProject PRJNA49810 by Mackenzie-Impoinvil et al., 2019.

## Funding

This work was supported by a 10.13039/100010269Wellcome Trust Senior Research Fellowship in Biomedical Sciences to CSW (101893/Z/13/Z and 217188/Z/19/Z) and from CDC's Advanced Molecular Detection (AMD) program.

## Authors contribution

Conceived and designed by CSW, AEL and MOK. LMI, carried out field collection of mosquitoes. MOK, LMI, SSI and AM carried out molecular analyses, with contribution from HI. MOK, SSI and JH conducted data analyses. MOK and SSI wrote the manuscript with contribution from all authors. All authors read and approved final version of the manuscript.

## Declaration of Competing Interest

The authors declare no competing or financial interests. The findings and conclusions in this paper are those of the authors and do not necessarily represent the official position of the Centers for Disease Control and Prevention (CDC).

## References

[bb0005] Bhatt S., Weiss D., Cameron E., Bisanzio D., Mappin B., Dalrymple U., Battle K., Moyes C., Henry A., Eckhoff P. (2015). The effect of malaria control on *Plasmodium falciparum* in Africa between 2000 and 2015. Nature.

[bb0010] Cingolani P., Platts A., Wang L.E., L., Coon, M., Nguyen, T., Wang, L., Land, S. J., LU, X. & Ruden, D. M. (2012). A program for annotating and predicting the effects of single nucleotide polymorphisms, SnpEff: SNPs in the genome of *Drosophila melanogaster* strain w1118; iso-2; iso-3. Fly (Austin).

[bb0015] Colovos C., Yeates T.O. (1993). Verification of protein structures: patterns of nonbonded atomic interactions. Protein Sci..

[bb0020] Garrison E., Marth G. (2012). Haplotype-based variant detection from short-read sequencing. *arXiv preprint arXiv:1207.3907*. https://arxiv.org/abs/1207.3907.

[bb0025] Garrison E., Kronenberg Z.N., Dawson E.T., Pedersen B.S., Prins P. (2021). Vcflib and tools for processing the Vcf variant call format. bioRxiv.

[bb0030] Gilbert L.I., Gill S.S. (2010). https://www.nhbs.com/insect-control-book.

[bb0035] Gotoh O. (1992). Substrate recognition sites in cytochrome P450 family 2 (CYP2) proteins inferred from comparative analyses of amino acid and coding nucleotide sequences. J. Biol. Chem..

[bb0040] Gotoh O. (1992). Substrate recognition sites in cytochrome P450 family 2 (CYP2) proteins inferred from comparative analyses of amino acid and coding nucleotide sequences. J. Biol. Chem..

[bb0045] Guengerich F.P., Martin M.V., Sohl C.D., Cheng Q. (2009). Measurement of cytochrome P450 and NADPH–cytochrome P450 reductase. Nat. Protoc..

[bb0050] Hancock P.A., Wiebe A., Gleave K.A., Bhatt S., Cameron E., Trett A., Weetman D., Smith D.L., Hemingway J., Coleman M., Gething P.W., Moyes C.L. (2018). Associated patterns of insecticide resistance in field populations of malaria vectors across Africa. Proc. Natl. Acad. Sci..

[bb0055] Hemingway J., Ranson H., Magill A., Kolaczinski J., Fornadel C., Gimnig J., Coetzee M., Simard F., Roch D.K., Hinzoumbe C.K., Pickett J., Schellenberg D., Gething P., Hoppé M., Hamon N. (2016). Averting a malaria disaster: will insecticide resistance derail malaria control?. Lancet (London, England).

[bb0065] Ibrahim S.S., Amvongo-Adjia N., Wondji M.J., Irving H., Riveron J.M., Wondji C.S. (2018). Pyrethroid resistance in the major malaria vector Anopheles funestus is exacerbated by overexpression and overactivity of the P450 CYP6AA1 across Africa. Genes.

[bb0070] Irwin J.J., Shoichet B.K. (2005). Zinc--a free database of commercially available compounds for virtual screening. J. Chem. Inf. Model..

[bb0075] Khambay B., Jewess P., Gilbert L.I., Iatrou K., Gill S.S. (2004). Pyrethroids. Comprehensive Molecular Insect Science.

[bb0080] Lange G. (2006). Pesticide Use in Rice Cultivation in Tarapoto, Peru. https://stud.epsilon.slu.se/11932/.

[bb0085] Laporta G.Z., Linton Y.-M., Wilkerson R.C., Bergo E.S., Nagaki S.S., Sant’ana, D. C. & Sallum, M. A. M. (2015). Malaria vectors in South America: current and future scenarios. Parasit. Vectors.

[bb0090] Li H. (2013). Aligning sequence reads, clone sequences and assembly contigs with BWA-MEM. *arXiv preprint arXiv:1303.3997*. https://arxiv.org/abs/1303.3997.

[bb0095] Lol J.C., Castellanos M.E., Liebman K.A., Lenhart A., Pennington P.M., Padilla N.R. (2013). Molecular evidence for historical presence of knock-down resistance in *Anopheles albimanus*, a key malaria vector in Latin America. Parasit. Vectors.

[bb0100] Lucas E.R., Miles A., Harding N.J., Clarkson C.S., Lawniczak M.K.N., Kwiatkowski D.P., Weetman D., Donnelly M.J. (2019). Whole-genome sequencing reveals high complexity of copy number variation at insecticide resistance loci in malaria mosquitoes. Genome Res..

[bb0105] Mackenzie-Impoinvil L., Weedall G.D., Lol J.C., Pinto J., Vizcaino L., Dzuris N., Riveron J., Padilla N., Wondji C., Lenhart A. (2019). Contrasting patterns of gene expression indicate differing pyrethroid resistance mechanisms across the range of the New World malaria vector *Anopheles albimanus*. PLoS One.

[bb0110] Miles A., Harding N.J., Bottà G., Clarkson C.S., Antão T., Kozak K., Schrider D.R., Kern A.D., Redmond S., Sharakhov I., Pearson R.D., Bergey C., Fontaine M.C., Donnelly M.J., Lawniczak M.K.N., Kwiatkowski D.P., Donnelly M.J., Ayala D., Besansky N.J., Burt A., Caputo B., Della Torre A., Fontaine M.C., Godfray H.C.J., Hahn M.W., Kern A.D., Kwiatkowski D.P., Lawniczak M.K.N., Midega J., Neafsey D.E., O’loughlin S., Pinto J., Riehle M.M., Sharakhov I., Vernick K.D., Weetman D., Wilding C.S., White B.J., Troco A.D., Pinto J., Diabaté A., O’loughlin S., Burt A., Costantini C., Rohatgi K.R., Besansky N.J., Elissa N., Pinto J., Coulibaly B., Riehle M.M., Vernick K.D., Pinto J., Dinis J., Midega J., Mbogo C., Bejon P., Wilding C.S., Weetman D., Mawejje H.D., Donnelly M.J., Weetman D., Wilding C.S., Donnelly M.J., Stalker J., Rockett K., Drury E., Mead D., Jeffreys A., Hubbart C., Rowlands K., Isaacs A.T., Jyothi D., Malangone C., Vauterin P., Jeffery B., Wright I., Hart L., Kluczyński K., Cornelius V., Macinnis B., Henrichs C., Giacomantonio R., Kwiatkowski D.P., The Anopheles Gambiae Genomes, C., Data Analysis, G., Partner Working, G., Sample, C. A., Burkina, F., Cameroon, Gabon, Guinea, Guinea, B., Kenya, Uganda, Crosses, Sequencing, Data, P., Web Application, D. & Project, C (2017). Genetic diversity of the African malaria vector *Anopheles gambiae*. Nature.

[bb0115] Mugenzi L.M.J., Menze B.D., Tchouakui M., Wondji M.J., Irving H., Tchoupo M., Hearn J., Weedall G.D., Riveron J.M., Wondji C.S. (2019). Cis-regulatory *CYP6P9b* P450 variants associated with loss of insecticide-treated bed net efficacy against *Anopheles funestus*. Nat. Commun..

[bb0120] Müller P., Warr E., Stevenson B.J., Pignatelli P.M., Morgan J.C., Steven A., Yawson A.E., Mitchell S.N., Ranson H., Hemingway J. (2008). Field-caught permethrin-resistant *Anopheles gambiae* overexpress *CYP6P3*, a P450 that metabolises pyrethroids. PLoS Genet..

[bb0125] Omura T., Sato R. (1964). The carbon monoxide-binding pigment of liver microsomes. I. evidence for its hemoprotein nature. J. Biol. Chem..

[bb0130] Orjuela L.I., Álvarez-Diaz D.A., Morales J.A., Grisales N., Ahumada M.L., Venegas H.J., Quiñones M.L., Yasnot M.F. (2019). Absence of knockdown mutations in pyrethroid and DDT resistant populations of the main malaria vectors in Colombia. Malar. J..

[bb0135] Poulos T.L., Finzel B.C., Gunsalus I.C., Wagner G.C., Kraut J. (1985). The 2.6-A crystal structure of *Pseudomonas putida* cytochrome P-450. J. Biol. Chem..

[bb0140] Pritchard M.P., Ossetian R., Li D.N., Henderson C.J., Burchell B., Wolf C.R., Friedberg T. (1997). A general strategy for the expression of recombinant human cytochrome P450s in *Escherichia coli* using bacterial signal peptides: expression of *CYP3A4*, *CYP2A6*, and *CYP2E1*. Arch. Biochem. Biophys..

[bb0145] Quiñones M.L., Norris D.E., Conn J.E., Moreno M., Burkot T.R., Bugoro H., Keven J.B., Cooper R., Yan G., Rosas A. (2015). Insecticide resistance in areas under investigation by the International Centers of Excellence for Malaria Research: a challenge for malaria control and elimination. Am. J. Trop. Med. Hygiene.

[bb0150] Riveron J.M., Irving H., Ndula M., Barnes K.G., Ibrahim S.S., Paine M.J., Wondji C.S. (2013). Directionally selected cytochrome P450 alleles are driving the spread of pyrethroid resistance in the major malaria vector *Anopheles funestus*. Proc. Natl. Acad. Sci..

[bb0155] Riveron J.M., Ibrahim S.S., Chanda E., Mzilahowa T., Cuamba N., Irving H., Barnes K.G., Ndula M., Wondji C.S. (2014). The highly polymorphic *CYP6M7* cytochrome P450 gene partners with the directionally selected *CYP6P9a* and *CYP6P9b* genes to expand the pyrethroid resistance front in the malaria vector *Anopheles funestus* in Africa. BMC Genomics.

[bb0160] Riveron J.M., Yunta C., Ibrahim S.S., Djouaka R., Irving H., Menze B.D., Ismail H.M., Hemingway J., Ranson H., Albert A., Wondji C.S. (2014). A single mutation in the *GSTe2* gene allows tracking of metabolically based insecticide resistance in a major malaria vector. Genome Biol..

[bb0165] Sali A., Blundell T.L. (1993). Comparative protein modelling by satisfaction of spatial restraints. J. Mol. Biol..

[bb0170] Schuler M.A., Berenbaum M.R. (2013). Structure and function of cytochrome P450s in insect adaptation to natural and synthetic toxins: insights gained from molecular modeling. J. Chem. Ecol..

[bb0175] Stevenson B.J., Bibby J., Pignatelli P., Muangnoicharoen S., O’neill P.M., Lian L.-Y., Müller P., Nikou D., Steven A., Hemingway J. (2011). Cytochrome P450 6M2 from the malaria vector *Anopheles gambiae* metabolizes pyrethroids: sequential metabolism of deltamethrin revealed. Insect Biochem. Mol. Biol..

[bb0180] Susanna D., Pratiwi D. (2022). Current status of insecticide resistance in malaria vectors in the Asian countries: a systematic review [version 2; peer review: 1 approved, 2 approved with reservations]. F1000Research.

[bb0185] Thomsen R., Christensen M.H. (2006). MolDock: a new technique for high-accuracy molecular docking. J. Med. Chem..

[bb0190] Weedall G.D., Mugenzi L.M., Menze B.D., Tchouakui M., Ibrahim S.S., Amvongo-Adjia N., Irving H., Wondji M.J., Tchoupo M., Djouaka R. (2019). A cytochrome P450 allele confers pyrethroid resistance on a major African malaria vector, reducing insecticide-treated bednet efficacy. Sci. Transl. Med..

[bb0195] WHO (2020). https://apps.who.int/iris/handle/10665/337660.

[bb0200] Wickham H. (2011).

[bb0205] Yano J.K., Wester M.R., Schoch G.A., Griffin K.J., Stout C.D., Johnson E.F. (2004). The structure of human microsomal cytochrome P450 3A4 determined by X-ray crystallography to 2.05-A resolution. J. Biol. Chem..

[bb0210] Yunta C., Hemmings K., Stevenson B., Koekemoer L.L., Matambo T., Pignatelli P., Voice M., Nász S., Paine M.J. (2019). Cross-resistance profiles of malaria mosquito P450s associated with pyrethroid resistance against WHO insecticides. Pestic. Biochem. Physiol..

