# Supplementary material for: Pyrethroid resistance in the New World malaria vector Anopheles albimanus is mediated by cytochrome P450 CYP6P5
Source: Pestic Biochem Physiol. 2022 May;183:105061. doi: 10.1016/j.pestbp.2022.105061 (PMC9125164; doi:10.1016/j.pestbp.2022.105061)
Supplement: Supplementary file 1 — Supplementary material 1 [file mmc8.docx]

**Table S1: Primers used for amplification and functional characterisation of *AaCYP6P5***

| **Primer** | **Sequence** |
| --- | --- |
| AaCYP6P5 forward | 5' ATGGAAACGATTAGTGTCTG 3' |
| AaCYP6P5 reverse | 5' tctagaTCATTTCGTGGCCACCTTC 3' |
| ompA+2 forward | 5' GgaattccatatgAAAAAGACAGCTATCGCG 3' |
| 3'ompA_5'AaCYP6P5 Reverse | 5‘ CAGACACTAATCGTTTCCATggagcggcctgcgctacgg 3' |
| pUAS_CYP6P5 forward | 5‘ agatctATGGAAACGATTAG 3' |
| pUAS_CYP6P5 reverse | 5‘ tctagaTCATTTCGTGGCCACC 3' |
| qPCRtrg_Albi6P5F | 5‘ GGATTTATCTTTCTCGTCTCGATC 3' |
| qPCRtrg_Albi6P5R | 5‘ TTCACGTGTCCGTAGAAGAAGT 3' |

**Table S2: Predicted binding parameters of the top-ranked docking mode of various insecticides in CYP6P5 model**

| **Insecticide** | **Rank** | **MolDock Score** | **Protein** | **Hydrogen bond** | **Docking score** | **Electrostatic short** | **Electrostatic**  **Long** |
| --- | --- | --- | --- | --- | --- | --- | --- |
| α-cypermethrin | 1^st^ | -157.5 | -133.6 | -7.5 | -154.3 | -8.2 | -1.32 |
| Deltamethrin | 1^st^ | -139 | -140.1 | -5.9 | -139.6 | 0 | -1.30 |
| Permethrin | 1^st^ | -137.6 | -124.1 | 0 | -137.6 | 0 | 0 |
| DDT | 1^st^ | -93.9 | -98.2 | 0 | -93.9 | 0 | 0 |

MolDock Score = docking score evaluated after post-processing; Protein = the interaction energy between the pose and the target receptor (protein); Docking score = docking score evaluated before post-processing; electrostatic short = short-range electrostatic protein-ligand interactions (r<4.5Å); and electrostatic long = long-range electrostatic protein-ligand interactions (r>4.5Å).

**Table S3: The pattern of the retention times for these metabolites are similar for both insecticides**

|  | **Substrate** | **M1** | **M2** | **M3** | **M4** |
| --- | --- | --- | --- | --- | --- |
| **α-cypermethrin** | 9.79 | 5.79 | 5.28 | 4.82 | 3.98 |
| **Deltamethrin** | 10.19 | 5.97 | 5.41 | 5.06 | 4.04 |

Numbers are retention times in min.
